# [¹⁸F]Fluspidine PET/CT imaging to assess postoperative pain-associated σ1 receptor expression in female rats under analgesia

**DOI:** 10.1186/s41747-025-00646-2

**Published:** 2025-11-04

**Authors:** Renée M. Girbig, Anne Rix, Jasmin Baier, Leonie Tix, Anna M. Hartmann, Wenjia Liu, Pascal Paschenda, Alexandru Florea, Masoud Sadeghzadeh, Karolin Becker, Rupert Palme, Felix M. Mottaghy, René Tolba, Fabian Kiessling

**Affiliations:** 1 Institute for Experimental Molecular Imaging, Faculty of Medicine, RWTH Aachen University, Aachen, Germany; 2 Institute for Laboratory Animal Science and Experimental Surgery, Faculty of Medicine, RWTH Aachen University, Aachen, Germany; 3 Department of Nuclear Medicine, University Hospital RWTH Aachen, Aachen, Germany; 4 Department of Radiology and Nuclear Medicine, Maastricht University Medical Center (MUMC+), Maastricht, Netherlands; 5 Unit of Physiology, Pathophysiology and Experimental Endocrinology, Department of Biomedical Sciences, University of Veterinary Medicine Vienna, Vienna, Austria; 6 GROW Research Institute for Oncology and reproduction, Maastricht University, Maastricht, Netherlands

**Keywords:** [18F]fluspidine, Pain measurement, Positron emission tomography computed tomography, Rats (Wistar), Sigma-1 receptor

## Abstract

**Background:**

Pain assessment in animal models is challenging, as behavioral tests often lack sensitivity. Particularly under analgesia, it is unclear whether pain occurs without medication. Imaging of pain-associated pathways, such as σ1 receptor (σ1R) expression, offers a promising approach to better understand underlying mechanisms. Therefore, this study evaluated [¹⁸F]fluspidine positron emission tomography/computed tomography (PET/CT) imaging for detecting σ1R-mediated pain after partial liver resection in rats.

**Materials and methods:**

Postoperative pain was assessed in eighteen female Wistar rats undergoing skin incision or partial liver resection. Nine untreated rats served as controls. Carprofen was administered for three consecutive days after surgery. PET/CT imaging was performed on postoperative days 1, 4, and 7. At each time point, organs and incision sites of three animals were harvested for histological analysis. Postoperative pain and welfare were monitored by observational score sheets, the Open Field test, Rat Grimace Scale, Von Frey test, fecal corticosterone metabolites, and hemograms.

**Results:**

Despite analgesic treatment, PET/CT and immunohistochemistry revealed elevated σ1R expression at the abdominal incision site on day 1 after partial liver resection in comparison to the other groups, likely due to the additional peritoneal opening. σ1R expression normalized by day 4. No behavioral indicators of pain or distress were observed, though mechanical hypersensitivity was detected on day 4 in all groups, likely due to carprofen side effects.

**Conclusion:**

[^18^F]Fluspidine PET/CT imaging sensitively detected postoperative pain-associated σ1R expression independent of analgesia. This imaging modality could remarkably refine pain monitoring, opening to further studies using different pain and analgesia models.

**Relevance statement:**

[¹⁸F]Fluspidine PET/CT imaging demonstrates high sensitivity in detecting pain-associated σ1R upregulation despite non-steroidal anti-inflammatory drug administration. This approach offers valuable insights for refining pain assessment, improving severity grading, and enhancing the reliability and translational value of preclinical pain models.

**Key Points:**

- PET/CT imaging with [^18^F]fluspidine sensitively detects pain-associated σ1R expression post-liver resection.
- Necessary analgesia interferes with some behavioral tests, limiting their reliability for pain assessment.
- [^18^F]Fluspidine detects peripheral σ1R upregulation despite non-steroidal anti-inflammatory drug analgesia.
- Imaging pain-associated receptors provides valuable insights for refining preclinical pain monitoring.

**Graphical Abstract:**

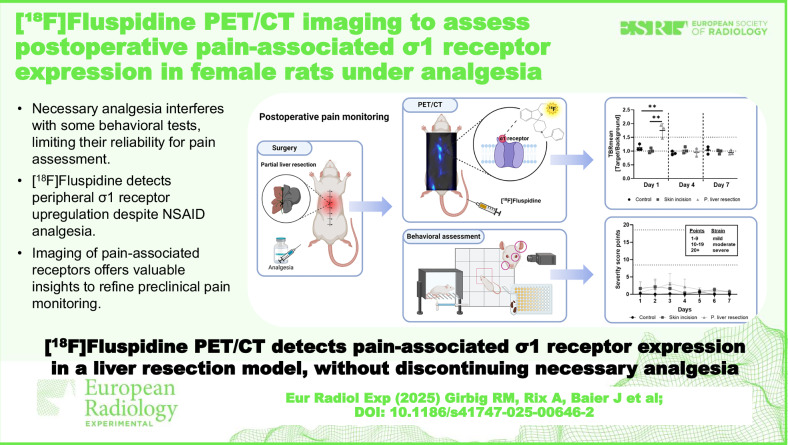

## Background

Despite enormous efforts in pain research, correctly detecting and localizing pain remains challenging. Especially in animals, the ability to accurately assess pain is limited since the most commonly used laboratory animals are flight animals, trying to hide pain from potential predators [1]. Ethically, appropriate pain management in research is essential to ensure animal welfare and to generate reliable data, as unrecognized pain can lead to biased results. Common pain assessment methods are indirect, such as monitoring activity, heart rate, or body weight. However, these can be influenced by factors unrelated to pain, like circadian rhythms, anxiety, handling, or individual behavior, leading to misinterpretation [2]. Moreover, behavioral or pain tests, often based on evoked response reflexes, also lack sensitivity when it comes to accurately detecting pain, as they depend on observers’ interpretation and can be influenced by experimental conditions [3, 4]. Furthermore, accurate assessment of the presence of pain is also essential for the refinement of analgesic treatments of laboratory animals. Although the use of analgesia is required by law for any potentially painful intervention, it is often difficult to assess whether the amount of given analgesia is appropriate. This can result in over-medication or inadequate pain relief, which can both alter the study outcome. With a more precise localization of pain transmission, analgesia could be administered locally and, thus, be better regulated in terms of duration and intensity. While the direct measurement of pain sensation in animals is not possible, developing novel approaches that move beyond traditional evoked-response testing—such as targeting specific receptors and transmission pathways—is essential for advancing translational pain research and improving laboratory animal welfare.

Alongside these preclinical challenges, clinical pain assessment in humans also faces significant limitations. Pain evaluation remains inherently subjective, influenced by psychological, cultural, and individual factors, and is often complicated by discrepancies between the perceived pain location and the actual site of tissue injury or lesion, posing substantial challenges for accurate diagnosis and treatment. These complexities further underscore the necessity for continued basic research that advances animal welfare while ultimately facilitating long-term clinical translation.

Over the past decade, molecular imaging has become an important tool in pain research, particularly by targeting specific pain-associated markers like the σ1 receptor (σ1R). This receptor is a ligand-regulated chaperone protein expressed in the mitochondria-associated endoplasmic reticulum membrane and plasma membranes of cells in the central and peripheral nervous systems [5]. It regulates protein folding, oxidative stress, and cell survival, while playing a key role in pain signaling pathways by modulating and interacting with neurotransmitter receptors, ion channels, and opioid receptors [6]. While peripheral nociceptors (*e.g.*, TRPV1, Nav1.7, and P2X3) detect harmful stimuli and initiate electrical signals, and central receptors (*e.g.*, NMDA and AMPA receptors) modulate synaptic transmission in the spinal cord and brain, the σ1R occupies a unique regulatory role, acting as a dynamic modulator of both peripheral and central pain processing mechanisms [5]. Its expression persists even under non-steroidal analgesics, which primarily interfere with the cyclooxygenase signaling pathway [7], thereby positioning the σ1R as an especially promising target for advancing pain research and monitoring pain expression.

The important role of the σ1R, upregulated during neuropathic and nociceptive pain [8–10], led to the development of [^18^F]fluspidine, as one of the potent positron emission tomography (PET) radiotracers (*K*_i_σ1 = 2.3 nM) that binds selectively to the receptor [11]. In this context, preclinical studies have shown promising results for [^18^F]fluspidine and similar tracers in detecting changes in σ1R expression associated with various pain conditions in animals [12, 13]. These findings underscore the importance of continued basic research on σ1R-targeting tracers, particularly in view of initial clinical data suggesting their potential for precise pain localization [14, 15] and therapeutic application [16].

So far, preclinical research on [^18^F]fluspidine has primarily focused on σ1R detection in the central nervous system [17–20]. However, further investigation of σ1R expression in the peripheral nervous system is crucial for gaining a deeper understanding of pain origin and transmission in various pain models [15]. Therefore, this study aimed to assess the suitability of this radiotracer in visualizing and localizing postoperative pain-associated σ1R expression in the peripheral system using an established partial liver resection rat model. Following the need for a valuable method to refine pain assessment and analgesia protocols, we used PET/computed tomography (CT) imaging to detect σ1R expression during and after analgesia and correlate the results with common behavioral pain assessment methods.

## Materials and methods

### Experimental setup

All animal experiments adhered to animal welfare legislation (German law for the protection of animals and Directive 2010/63/EU) [21]. The full ethical proposal was approved by the responsible authority (LANUV NRW–“Landesamt für Natur, Umwelt und Verbraucherschutz Nordrhein-Westfalen”, Recklinghausen, Germany, AZ: 84-02.04.2017.A304). All animals in the present study received human care according to the principles of the “Guide for the Care and Use of Laboratory Animals” (8th edition, NIH Publication, 2011, USA).

**Fig. 1:**
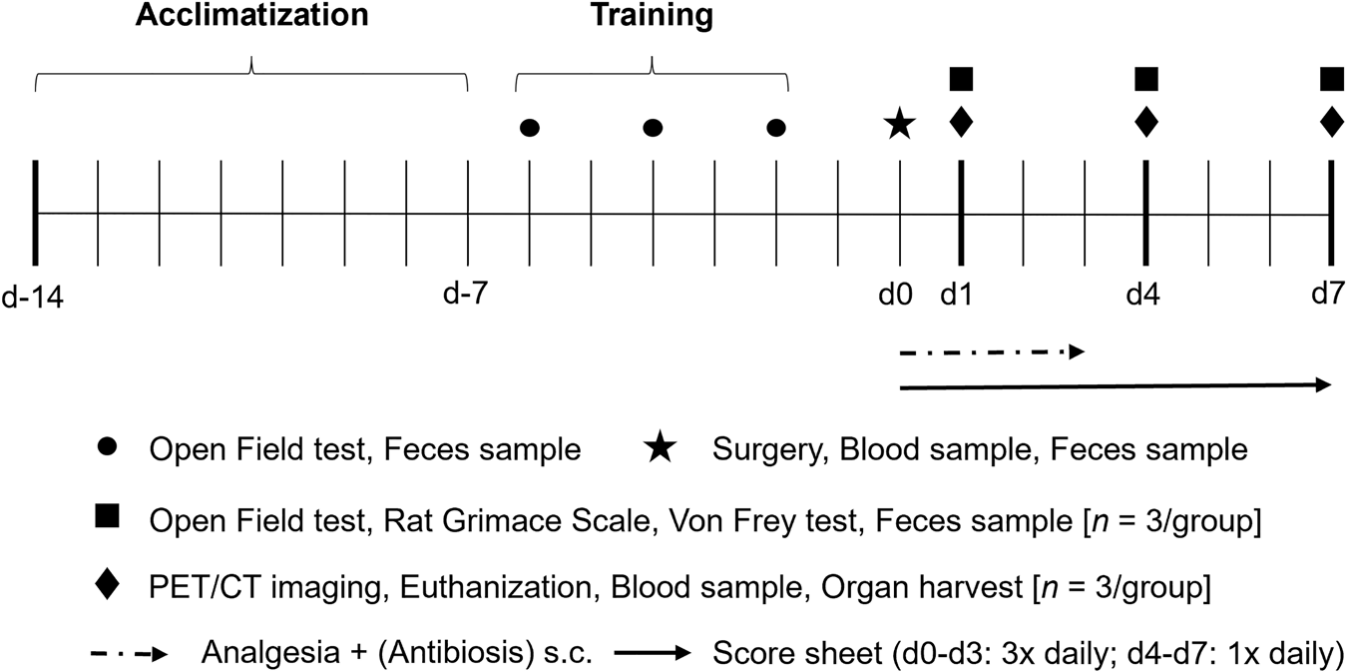
Experimental timeline for monitoring postoperative σ1R expression via PET/CT imaging and behavioral testing in a partial liver resection rat model. Following a training phase, rats underwent either a skin incision or a partial liver resection. PET/CT imaging with [^18^F]fluspidine was conducted on postoperative days 1, 4, and 7 alongside behavioral assessments. Three animals were evaluated at each time point. Analgesia was administered daily for three consecutive days post-surgery to all groups (including the control group). Surgery groups additionally received antibiotics to prevent infection. PET/CT, Positron emission tomography/computed tomography

First, the σ1R binding specificity of the in-house synthesized [^18^F]fluspidine was confirmed through autoradiography, followed by *in vivo* biodistribution studies in healthy female Wistar rats (*n* = 3). To evaluate postoperative pain, rats (age: 6–8 weeks) were randomly assigned to three groups: skin incision (*n* = 9), partial liver resection (*n* = 9), and control (*n* = 9). All animals received analgesia (carprofen) for three consecutive days post-surgery; surgical groups additionally received antibiotics. To ensure comparability between groups, control animals were also anesthetized and treated with analgesia for the same duration. PET/CT scans with [^18^F]fluspidine were performed on days 1, 4, and 7 after surgery to assess σ1R expression during and after analgesia (Fig. 1). On each of these days, prior to imaging, behavioral indicators of pain or distress were assessed using a clinical score sheet (Supplemental Table S4), the Open Field test, the Rat Grimace Scale, and the Von Frey test (VFT). All animals had undergone three presurgical training sessions for the Open Field test.

Following each PET/CT scan, three animals per group were euthanized for tissue collection (skin, muscle, and major organs) for histological analysis. Additionally, fecal samples were collected on intervention days to monitor stress hormone levels, and hemograms were obtained during surgery and euthanasia. To ensure reproducibility, detailed methodological information on radiotracer synthesis [11, 22, 23], autoradiography [12, 20], biodistribution, PET/CT imaging, animal experiments [21, 24–26], surgery [27], behavioral assessments [27–31], immunohistochemistry, and statistical data analysis is provided as Supplemental Material.

## Results

Exact values for all data points are provided in the Supplementary Tables referenced throughout the Results section.

### *In vitro* autoradiography and *in vivo* metabolism study

σ1R binding specificity of [^18^F]fluspidine was assessed by autoradiography. Preblocked brain slices showed significantly lower radiotracer uptake (Haloperidol: *p* < 0.001; Fluspidine: *p* = 0.024) compared to nonblocked slices (Supplemental Fig. S2 and Table S1), confirming binding specificity. *In vivo* biodistribution analysis in healthy rats demonstrated that [^18^F]fluspidine rapidly distributed throughout the body after injection. Blood tracer concentration decreased significantly, from (30.6–46.5%) 5 min after injection, with most of the unbound tracer cleared from the blood circulation within 60 min (< 8%) (Supplemental Table S2). After 60 mins, the unbound radiotracer accumulated in the bladder and liver, while free fluorine was observed in the bones.

### Postoperative σ1R imaging with PET/CT

**Fig. 2:**
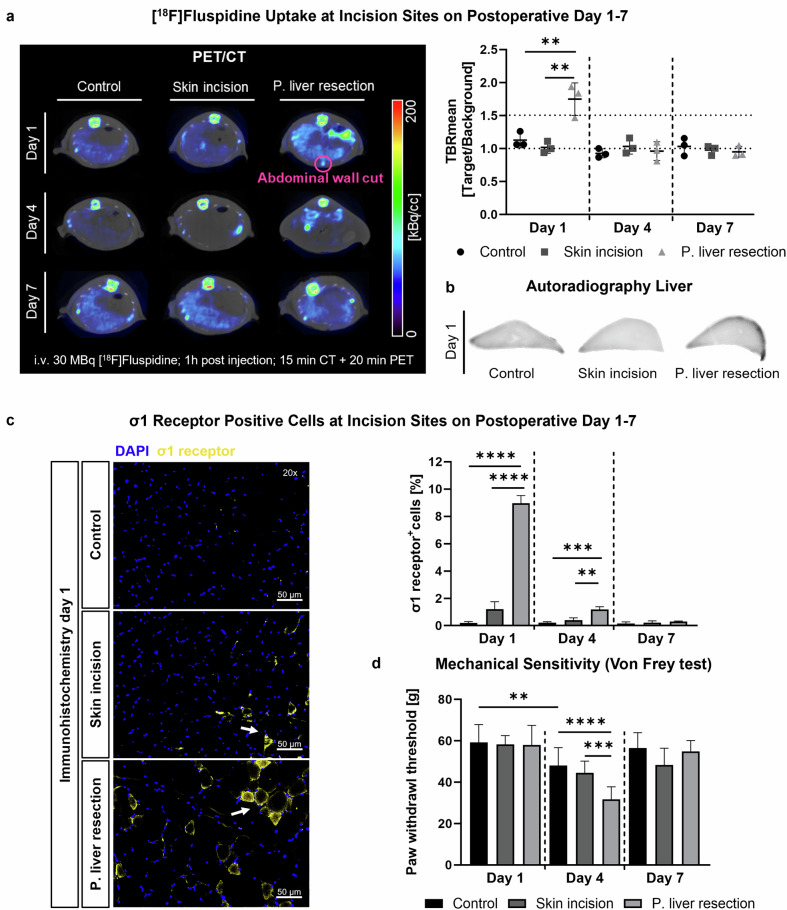
PET/CT quantification and immunohistochemical validation of [^18^F]fluspidine uptake at incision sites on postoperative day 1–7. **a** PET/CT images and quantitative analysis show significantly higher radiotracer uptake at incision sites after partial liver resection compared to other groups on day 1. **b** Liver capsule tracer uptake is detected by autoradiography in liver-resected rats. **c** Significantly more σ1R^+^ cells (white arrow) are detected by immunohistochemical staining at incision sites after partial liver resection compared to other groups on day 1. **d** Paw withdrawal threshold is reduced on day 4 in all groups, especially in rats that underwent liver resection. Means ± standard deviations. Scale bar: 50 μm; 20× magnification. i.v., Intravenous; P, Partial. **p < 0.01, ***p < 0.0005, ****p < 0.0001

Postoperative [^18^F]fluspidine uptake in the abdominal wound area of partial liver resected rats on day 1 was significantly higher than in the skin incision (*p* = 0.009) and control groups (*p* = 0.004; Fig. 2a). Liver capsule uptake was not detectable by PET/CT but was identified post-mortem at the resection site by autoradiography (Fig. 2b). On days 4 and 7, no significant tracer uptake was observed in the wound area (post liver resection: d4 *p* = 0.733; d7 *p* = 0.944; skin incision: d4 *p* = 0.531; d7 *p* = 0.788; Fig. 2a and Supplemental Figs. S5–S7). PET/CT findings correlated with immunohistochemical stainings, showing a higher percentage of σ1R-positive (σ1R^+^) cells on day 1 in the liver resection group compared to the other groups (skin incision: *p* < 0.001; control: *p* < 0.001; Fig. 2c, Supplemental Fig. S3, and Table S3). On day 4, residual σ1R^+^ cells were detected in liver resected rats (skin incision: *p* < 0.001; control: *p* < 0.002), with their quantity likely below the current detection threshold of PET/CT. By day 7, the percentage of σ1R^+^ cells in the surgical groups was comparable to the control group (*p* = 0.309). TUNEL staining indicated a higher percentage of apoptotic cells in the liver resection group compared to skin incision (*p* = 0.019) and control groups (*p* = 0.002). By days 4 and 7, apoptosis rates were comparable between all groups (d4 *p* = 0.643; d7 *p* = 0.591; Supplemental Fig. S4 and Table S3).

### Postoperative pain monitored by physiological parameters

**Fig. 3:**
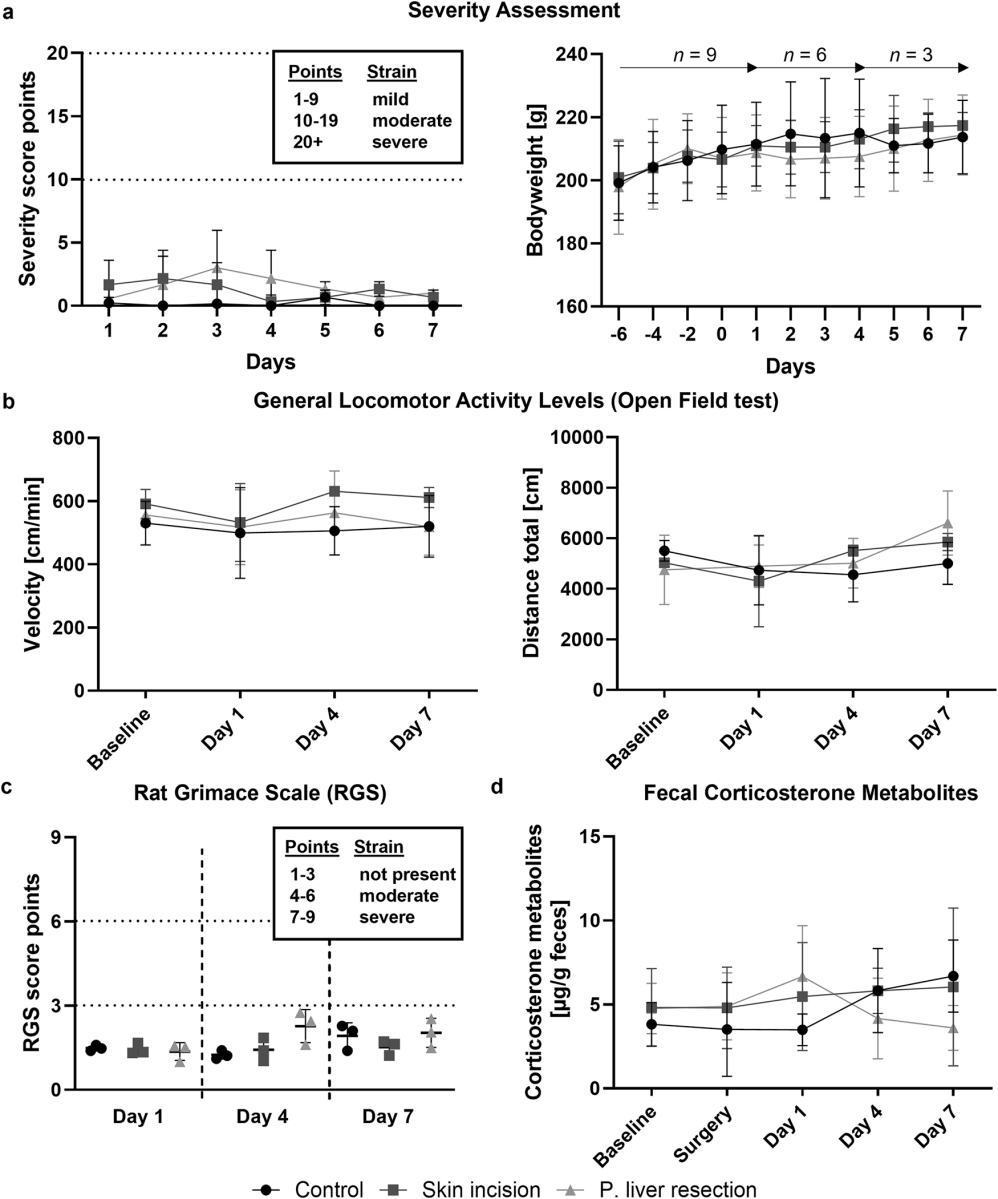
Postoperative pain monitoring by physiological parameters. **a** Score sheet evaluation (*i.e*., bodyweight, general condition, spontaneous behavior, clinical symptoms) indicates a mild burden in all groups. Bodyweight remained stable in all groups. **b** General locomotor activity (Open Field test) is not affected by surgery. **c** Rat Grimace Scale does not show any significant differences between the groups. **d** Levels of fecal corticosterone metabolites do not change over time. P, Partial

Daily postoperative welfare scoring using an adapted clinical score sheet (Supplemental Table S4) showed that all groups remained within a mild severity range (1–9 points) throughout the experiment (*p* = 0.258; Fig. 3a). Consistently, bodyweights remained stable after surgery (*p* = 0.998; Fig. 3a). Motor activity, including velocity and total distance moved, did not differ significantly from baseline values (velocity: *p* = 0.322; distance: *p* = 0.178, Fig. 3b and Supplemental Table S5). Evaluation of the Rat Grimace Scale showed no elevated scores post-surgery (*p* = 0.378, Fig. 3c and Supplemental Table S5). Furthermore, stress-associated levels of fecal corticosterone metabolite values were comparable to baseline values in all groups (*p* = 0.456, Fig. 3d and Supplemental Table S5). The same holds true for the paw withdrawal threshold on postoperative day 1 (*p* = 0.986; Fig. 2d and Supplemental Table S5).

However, on day 4, the first day without analgesia, partial liver resected rats showed a significantly lower paw threshold compared to controls (*p* < 0.001), consistent with the residual percentage of σ1R^+^ cells (Fig. 2c). Interestingly, the control group also showed a significantly lower paw threshold on day 4 compared to the control group on day 1 (*p* = 0.002). On day 7, all groups showed recovery to baseline values (*p* = 0.437). Except for a slight reduction in hematocrit and hemoglobin levels in liver resected animals on day 1, hemograms showed consistent erythrocyte, thrombocyte, hemoglobin, and hematocrit values throughout the experiment (Supplemental Table S6). However, leukocyte counts decreased significantly in all groups (post-liver resection: d1 *p* = 0.043, d4 *p* = 0.047, d7 *p* = 0.022; skin incision: d1 *p* = 0.031, d4 *p* = 0.005, d7 *p* = 0.041; control d1 *p* = 0.031, d4 *p* = 0.003, d7 *p* = 0.026), regardless of the intervention and the postoperative day (Supplemental Fig. S8).

## Discussion

Molecular imaging offers a promising approach to monitor and localize pain transmission in laboratory animals, overcoming some possible limitations of traditional evoked response tests, which can be influenced by non-pain-related factors. Unlike these methods, molecular imaging directly visualizes pain-associated receptor expression, independently of analgesic treatment. In this context, [^18^F]fluspidine, has shown potential for detecting pain-related changes in σ1R expression. However, research has primarily focused on the central nervous system, and further investigation of the peripheral nervous system is needed to refine analgesia protocols. Therefore, this study evaluated [^18^F]fluspidine PET/CT for assessing peripheral σ1R expression in a postoperative rat model of partial liver resection.

The binding specificity of [^18^F]fluspidine to the σ1R, previously validated in other studies [11, 32], was tested by autoradiography using rat brain slices, which are known for their high density of σ1Rs [33, 34]. As expected, pre-blocked slices treated with haloperidol, a common σ1R antagonist [35, 36], showed significantly lower tracer uptake compared to non-blocked slices, confirming the specificity of the radiotracer. *In vivo* metabolism study in healthy rats demonstrated that [^18^F]fluspidine was rapidly distributed throughout the body, with approximately 70% of the unbound tracer cleared from the circulation within 5 min [37]. After this time, the unbound tracer accumulated predominantly in the bladder and liver, indicating metabolic degradation. High renal excretion has been reported previously [11], with faster excretion in female rats probably due to hormonal and anatomical differences [38]. The observed rapid liver degradation is also consistent with results from studies using rat liver microsomes [39]. Moreover, we detected free fluorine in the bones, particularly in the spine and ribs. Defluorination and the incorporation of free fluoride-18 into bones, forming fluoroapatite, is common with most ^18^F-labeled radiotracers. Previous studies in rats have reported high uptake of free fluoride-18 in trabecular bones 60 min post-injection, likely due to high perfusion and osteoblastic activity [40]. However, this did not affect our results as the focus was on the abdominal wound area, and this phenomenon is restricted to bones.

Postoperative PET/CT imaging with [^18^F]fluspidine successfully localized σ1R expression in the abdominal wound area of analgesized liver resected rats on postoperative day 1. No significant radiotracer uptake was observed in the surgical groups on the subsequent days. Interestingly, tracer uptake was prominent in the liver resected animals but absent in the skin incision group. This difference may be due to the additional opening of the peritoneum during liver resection, as the visceral peritoneum, innervated by visceroafferent fibers associated with sympathetic nerves, has higher expression density of σ1Rs than the upper skin layers [41], as shown by immunohistochemistry. Although partial liver resection was performed, PET/CT did not detect σ1R upregulation in the innervated liver capsule, probably due to strong hepatic metabolism and respiratory motion. However, as expected, post-mortem autoradiography revealed higher [^18^F]fluspidine uptake at the resection site in the innervated liver capsule compared to control liver tissue. Immunohistochemical stainings of the abdominal wound area confirmed our PET/CT findings, showing only a few residual σ1R^+^ cells on postoperative day 4 in liver resected rats, with numbers too low to be detected by PET/CT.

Notably, overall σ1R upregulation post-surgery was much lower than under ischemic conditions [42], highlighting the sensitivity of our approach to detect even low levels of σ1R expression in this moderate pain model. Moreover, our findings suggest that the observed σ1R expression is closely linked to wound healing processes. In rats, wound healing of skin incisions follows three main phases—inflammation, proliferation and maturation/remodeling—and is largely completed by day 7 [43]. Inflammation peaks within the first three days, involving apoptotic processes and cytokine pathways, which are also linked to σ1R expression [44, 45]. This might explain the downregulation of σ1R expression as seen in immunohistochemistry, which resulted in a lack of enhanced uptake in PET/CT imaging on day 4, after acute inflammation had subsided. Additionally, fewer apoptotic cells were detected in the wound area by day 4, confirming the end of the acute inflammatory phase. The administration of anti-inflammatory antibiotics, standard in such surgeries, likely contributed to wound healing and further downregulated σ1R expression, as these medications improve recovery and minimize postsurgical inflammation [46].

Despite the observed σ1R upregulation in liver-resected rats on postoperative day 1, no signs of welfare impairment were detected in any group regarding motor activity, the Rat Grimace Scale, VFT, hemograms, or fecal corticosterone metabolites. After discontinuing analgesia, VFT results showed hypersensitivity in liver-resected animals, consistent with the immunohistochemical findings of residual σ1R upregulation. However, the control group also exhibited significant hypersensitivity, indicating a possible experimental bias. The observed hypersensitivity is likely due to the long-term administration of carprofen, a non-steroidal anti-inflammatory drug (NSAID) known to induce mechanical hypersensitivity even in healthy animals when used at higher doses or for longer periods [47, 48]. Research has also shown that transient receptor potential (TRP) channels, which are critical for pain perception, can attenuate hypersensitivity when activated in combination with NSAIDs [47]. Since the σ1R regulates TRP calcium channels [49], the residual σ1R expression detected by immunohistochemistry on day 4 may have resulted in sustained TRP channel activation, which, in combination with carprofen administration, may have attenuated a possible hypersensitivity observed in the surgical groups. Notably, the surgery did not significantly affect the rats’ hemograms, except for a slight decrease in hematocrit and hemoglobin levels in liver-resected rats on day 1, which is typical after such interventions and likely due to blood loss during surgery [27]. However, a significant reduction in leukocytes was observed across all groups on the final day of the experiment compared to the day of surgery, independent of the postoperative day. Although cefuroxime is known to reduce leukocyte levels, it was not administered to the control animals, making this effect more likely due to gamma radiation exposure following radiotracer injection [50].

While this study presents promising findings in σ1R imaging and pain research, demonstrating [^18^F]fluspidine PET detection of peripheral σ1R upregulation under NSAID analgesia, certain limitations should be acknowledged. First, the number of animals was intentionally kept low to comply with the 3Rs principle and to conduct an initial feasibility assessment of the tracer in a surgical pain model, using only one sex as a pilot approach. Although this limits statistical power, it allowed for an ethical and focused exploration of tracer applicability, providing a solid foundation for future studies involving larger cohorts and different pain models. While initial research suggests that male and female rats respond similarly to partial liver resection in terms of behavioral testing and physiological parameters, previous preclinical studies have predominantly involved male rats [51]. This study aims to address this sex gap by expanding the limited data on female rats, acknowledging sex as a crucial biological variable for translational relevance [52]. Although some sex hormones, such as progesterone, estrogen, and dehydroepiandrosterone, have been reported to interact with the σ1R, the precise mechanisms of receptor activation and function, as well as the physiological relevance of these interactions, require further elucidation [53, 54]. Nevertheless, pain mechanisms can differ between sexes due to specific physiological characteristics, and overall hormone status remains an important factor in pain research. Therefore, future investigations should include both sexes. Furthermore, due to species differences and the complexity of human hormonal regulation, direct extrapolation of these findings to humans requires further validation. Second, to date, there is no known method to directly measure pain sensation in animals. Instead, [¹⁸F]fluspidine visualizes a specific aspect of the complex pain transmission system, particularly regarding spatial localization and temporal dynamics. Despite this limitation, the method offers valuable insights that could support future refinement of animal models by optimizing analgesic strategies (in both location and duration) and aiding in the selection of the most appropriate behavioral tests for specific models. Finally, due to its cost and complexity, this technique is not suited for routine use in all animal experiments and should not replace behavioral tests. However, its strategic application could provide high-impact data for model development or validation (*e.g.*, severity grading), refinement, and the ethical advancement of preclinical research.

In conclusion, we successfully demonstrated that [^18^F]fluspidine effectively detects peripheral σ1R upregulation, even under NSAID analgesia, in a model of moderate postoperative pain. The VFT showed sensitivity for pain detection, but was probably biased by analgesia, while other behavioral tests, likely being less sensitive to mild stress, did not show significant results. In this regard, our approach of monitoring pain-associated receptor expression and localizing upregulation without discontinuing necessary analgesia could potentially support the severity grading of new animal models, the re-evaluation of established models, and the optimization of analgesia protocols in the future. It may also serve as a reference for the selection of the most suitable behavioral or pain test. Beyond these possible applications, this approach also provides insight into pain-related patterns of σ1R expression, which could, in the future, also complement clinical assessments by offering a more mechanistic basis for understanding pain localization. However, as this was a pilot study, future experiments should include larger animal numbers and both sexes to strengthen the findings. Further research is also needed to assess the tracer’s applicability across different pain models.

## Supplementary information

Supplementary Information
**Supplemental Figure S1**. Schematic representation of the liver lobes resected during partial (50%) liver resection. The respective lobes, including the left part of the medial liver lobe (LML), the left lateral lobe (LLL), and both caudate lobes (CL), were ligated with 4/0 silk at the hilus and excised with scissors. To prevent bleeding, the remaining stumps were cauterized with bipolar forceps. LLL left lateral lobe; LML left median lobe; RML right median lobe; RLL right liver lobes; IRL inferior right lobe; SRL superior right lobe; CL caudate lobes; ACL anterior caudal lobe; PCL posterior caudal lobe. **Supplemental Table S1**. [^18^F]Fluspidine autoradiography blocking study. Detailed results on tracer uptake [prop.to.cnts/sec] in healthy rat brains. All results are reported as means ± standard deviations. **Supplemental Table S2**. [^18^F]Fluspidine *in vivo* metabolism study. Detailed results on tracer concentrations in blood and urine at 5-, 30-, and 60-min post-injection. **Supplemental Table S3**. PET/CT quantification and immunohistochemical (IHC) validation of [^18^F]fluspidine uptake at incision sites on postoperative day 1–7. Detailed information on radiotracer uptake and IHC staining for σ1 receptor positive (σ1R+) and apoptotic cells at the incision site. All results are reported as means ± standard deviations. **Supplemental Table S4**. Daily postoperative clinical score sheet monitoring in rats. Alterations in bodyweight, general state, spontaneous behavior, and surgery specific parameters were documented and allocated to a point grading system. No alterations in physiological state were graded as 0 points, whereas ≥ 20 points marked the highest severity and humane endpoint. **Supplemental Table S5**. Postoperative pain monitoring by physiological parameters. Detailed results of animal welfare assessment parameters (clinical score sheet, bodyweight, Open Field test: distance and velocity, Von Frey test: Paw withdrawl threshold, Rat Grimace Scale, Fecal corticosterone metabolite levels) of rats on postoperative day 1–7. FCMs, Fecal corticosterone metabolites; RGS, Rat Grimace Scale. All results are reported as means ± standard deviations. **Supplemental Table S6**. Hemogram results on surgery day and euthanisation day. Detailed results from hemogram analysis (leukocyte-, erythrocyte-, thrombocyte counts, hemoglobin, hematocrit) of all groups. All results are reported as means ± standard deviations. **Supplemental Figure S2**. [18F]Fluspidine autoradiography blocking study. (a-b) The radiotracer uptake in pre-blocked (haloperidol 10 μM or fluspidine 10 μM) rat brain slices is significantly lower than in non-blocked slices. n = 9; **p > 0.01, ***p > 0.0005. **Supplemental Figure S3**. Immunohistochemical (IHC) staining of the σ1 receptor at incision sites (skin and muscle) on postoperative day 1–7. Significantly more σ1 receptor positive cells (white arrow) are detected by IHC staining at the incision sites after liver resection compared to the control and skin incision group on postoperative day 1. On postoperative day 4, only a few residual σ1 receptor positive cells can be observed in liver resected rats. Comparable numbers of σ1 receptor positive cells can be detected on postoperative day 7 in all groups. Results are presented as means ± standard deviations. Scale bar: 50 μm (images taken at 20x magnification). P. partial. **p > 0.01, ***p > 0.0005, ****p > 0.0001. **Supplemental Figure S4**. Immunohistochemical (IHC) staining of apoptotic cells at incision sites (skin and muscle) on postoperative day 1–7. Significantly more TUNEL positive cells (white arrow) are detected by IHC staining at the incision sites after liver resection compared to the control and skin incision group on postoperative day 1. Comparable numbers of apoptotic cells can be detected on postoperative day 4 and 7 in all groups. Results are presented as means ± standard deviations. Scale bar: 50 μm (images taken at 20x magnification). P. partial. *p > 0.05, **p > 0.01. **Supplemental Figure S6**. PET/CT images of [18F]fluspidine uptake at incision sites on postoperative day 4. PET/CT images show no significant radiotracer uptake at the incision sites after surgery on postoperative day 4. Free fluorine is observed in the spine and ribs. PET/CT imaging was performed 60 min post-injection. ca caudal; cr cranial; d dorsal; P. partial; t transversal; v ventral. **Supplemental Figure S7**. PET/CT images of [^18^F]fluspidine uptake at incision sites on postoperative day 7. On postoperative day 7 no significant radiotracer uptake can be detected at the incision sites by PET/CT imaging. Free fluorine is observed in the spine and ribs. PET/CT imaging was performed 60 min post-injection. ca caudal; cr cranial; d dorsal; P. partial; t transversal; v ventral. **Supplemental Figure S8**. Leukocyte counts before surgery and on postoperative day 1–7. A significant decrease in leukocyte counts can be observed across all groups on the final day of the experiment (2 hours after [18F]fluspidine tracer injection), independent of the postoperative day or surgical intervention. P partial. *p > 0.05, **p > 0.01.

## Data Availability

The datasets generated during and/or analyzed during the current study are available from the corresponding author on reasonable request.

## Abbreviations

CT: Computed tomography
NSAID: Non-steroidal anti-inflammatory drug
PET: Positron emission tomography
TRP: Transient receptor potential
VFT: Von Frey test
σ1R: σ1 receptor
