# Supplementary material for: [¹⁸F]Fluspidine PET/CT imaging to assess postoperative pain-associated σ1 receptor expression in female rats under analgesia
Source: Eur Radiol Exp. 2025 Nov 4;9:108. doi: 10.1186/s41747-025-00646-2 (PMC12586836; doi:10.1186/s41747-025-00646-2)
Supplement: Supplementary file 1 — Supplementary Information Supplemental Figure S1. Schematic representation of the liver lobes resected during partial (50%) liver resection. The respective lobes, including the left part of the medial liver lobe (LML), the left lateral lobe (LLL), and both caudate lobes (CL), were ligated with 4/0 silk at the hilus and excised with scissors. To prevent bleeding, the remaining stumps were cauterized with bipolar forceps. LLL left lateral lobe; LML left median lobe; RML right median lobe; RLL right liver lobes; IRL inferior right lobe; SRL superior right lobe; CL caudate lobes; ACL anterior caudal lobe; PCL posterior caudal lobe. Supplemental Table S1. [18F]Fluspidine autoradiography blocking study. Detailed results on tracer uptake [prop.to.cnts/sec] in healthy rat brains. All results are reported as means ± standard deviations. Supplemental Table S2. [18F]Fluspidine in vivo metabolism study. Detailed results on tracer concentrations in blood and urine at 5-, 30-, and 60-min post-injection. Supplemental Table S3. PET/CT quantification and immunohistochemical (IHC) validation of [18F]fluspidine uptake at incision sites on postoperative day 1–7. Detailed information on radiotracer uptake and IHC staining for σ1 receptor positive (σ1R+) and apoptotic cells at the incision site. All results are reported as means ± standard deviations. Supplemental Table S4. Daily postoperative clinical score sheet monitoring in rats. Alterations in bodyweight, general state, spontaneous behavior, and surgery specific parameters were documented and allocated to a point grading system. No alterations in physiological state were graded as 0 points, whereas ≥ 20 points marked the highest severity and humane endpoint. Supplemental Table S5. Postoperative pain monitoring by physiological parameters. Detailed results of animal welfare assessment parameters (clinical score sheet, bodyweight, Open Field test: distance and velocity, Von Frey test: Paw withdrawl threshold, Rat Grimace Scale, Fecal [file 41747_2025_646_MOESM1_ESM.pdf]

[^{18}F]Fluspidine PET/CT imaging to assess postoperative pain-associated σ1 receptor expression in female rats under analgesia

ELECTRONIC SUPPLEMENTARY MATERIAL

Supplemental methodology information

Radiosynthesis of (-)-[^{18}F]fluspidine

(-)-[^{18}F]Fluspidine was synthesized from (-)-fluspidine and its precursor (ABX - advanced biochemical compounds, Radeberg, Germany) using the same radiolabeling conditions as described previously [*11,22,23*]. Identity and radiochemical purity were confirmed by radio-high pressure liquid chromatography (radio-HPLC) analyses with the non-radioactive compound and by radio-TLC (CH_2Cl_2/MeOH 9:1). Further information on radiochemical instruments and methods can be found below. For simplicity, the radioactive labeled tracer will be referred to as [^{18}F]fluspidine in the manuscript.

Radiochemistry-Instruments and general methods

Radio thin layer chromatography (radio-TLC) was performed using silica gel plates (Polygram® SIL G/UV254), pre-coated with a mixture of CH_2Cl_2/MeOH 9:1 (v/v) as eluent. The plates were exposed to storage phosphor screens (BAS-MS2025, FUJIFILM Co., Tokyo, Japan) and imaged with a Typhoon FLA7000 Fluorescent Image Analyzer (GE Healthcare Biosciences AB, Uppsala, Sweden). Image quantification was performed using ImageQuant TL8.1 software (GE Healthcare Life Sciences). No-carrier-added [^{18}F]fluoride was obtained in aqueous K[^{18}F]F state from the Research center Jülich (INM-5, Jülich, Germany). Analytical HPLC was performed using either a Knauer Smartline (KNAUER Advanced Scientific Instruments, Berlin, Germany) system, consisting of a Manager 5000, pump 1000, and UV-2600 diode array detector (190-510 nm), or a JASCO LC-2000 system with a PU-2080Plus pump, AS-2055Plus auto-injector (100 µL sample loop), and UV-2070Plus detector. Both systems were coupled to a gamma-radioactivity HPLC detector (Gabi Star, Elysia-Raytest GmbH, Straubenhardt, Germany). Data was processed using either GINA-Star software (v5.9, Elysia-Raytest GmbH) or Galaxie chromatography software (Agilent Technologies), with chromatograms recorded at 254 nm. For HPLC, either a LiChrospher 100 RP-C18 column (250 × 4.6 mm, 5 µm; CS-Chromatographie-Service, Langewehe, Germany) or a Reprosil-Pur 120 C18-AQ column (250 × 4.6 mm, 5 µm; Dr. Maisch HPLC GmbH, Germany) was used. The

eluent system comprised acetonitrile and 20 mM aqueous ammonium acetate (ACN/20 mM NH_4OAc aq., v/v, pH 6.8) at a flow rate of 1.0 mL/min. The gradient profile was as follows: 0–5 min, 100% eluent A (10% ACN/20 mM NH_4OAc aq.); 5–30 min, linear increase to 100% eluent B (90% ACN/20 mM NH_4OAc aq.); 30–33 min, 100% eluent B; 33–34 min, linear return to 100% eluent A; 34–40 min, 100% eluent A. The stated concentration of 20 mM NH_4OAc refers to the aqueous component of the eluent.

In vitro Autoradiography: Blocking Study

To ensure the σ1 receptor (σ1R) binding specificity of our in-house synthesized [^{18}F]Fluspidine radiotracer, we performed an autoradiography blocking study using paraffin-embedded rat brain tissue, as specific brain regions are known to have high σ1R density [*12,20*]. The brain slices were obtained post-mortem from control rats of our study cohort, all of which were female animals. The 20 µm sections were first deparaffinized and then preincubated with ethanol in 0.9 NaCl (n = 9), haloperidol (10 µM; n = 9), or fluspidine (10 µM; n = 9) (60 min, RT, humidified chamber). Subsequently, the slices were incubated with [^{18}F]fluspidine for 60 min, washed, and exposed to a phosphor image plate for 120 min. Afterwards, the plate was scanned using a BAS5000 reader (Fujifilm, Dielsdorf, Switzerland).

Animals and ethical statement

All animal experiments adhered to animal welfare legislation (German law for the protection of animals and Directive 2010/63/EU) [*21*]. The full ethical proposal was approved by the responsible authority (LANUV NRW–"Landesamt für Natur, Umwelt und Verbraucherschutz Nordrhein-Westfalen", Recklinghausen, Germany, AZ: 84-02.04.2017.A304). All animals in the present study received human care according to the principles of the "Guide for the Care and Use of Laboratory Animals" (8th edition, NIH Publication, 2011, USA). Thirty-three female Wistar rats (Janvier S.A.S., Saint-Berthevin Cedex, France; age: 6−8 weeks; weight: 150−175 g) were group-housed in filter-top cages (Type 2000, Tecniplast, Buguggiate, Italy) under specific pathogen-free conditions with controlled environmental factors (12-hour light/dark cycle, 22°C ± 2°C temperature, 30−70% relative humidity, red enrichment tube) following FELASA recommendations [*24*]. The rats had unrestricted access to a standard diet (rat/mouse maintenance #V1534-300, 10 mm; ssniff Spezialdiäten GmbH, Soest, Germany) and acidified drinking water ad libitum. Postoperative pain management complied with the Recommendations of the German Society for Laboratory Animal Science and the Initiative Veterinary Pain Therapy guidelines [*25*]. All efforts were made to minimize the number of animals used and their suffering according to the 3R Principles. Female rats were selected to expand the dataset in this research field, which has historically focused on males. Although hormonal status can influence pain transmission and associated receptors, current evidence

suggests that sex hormones have limited impact on σ1R activation or function. To minimize additional stress, the hormonal status of the animals (6−8 weeks old, peripubertal to sexually mature) was not assessed, as this study was not designed to investigate sex-specific differences. Pregnancy was excluded by housing females with same-sex cage mates and through visual inspection upon arrival from the breeder and during handling. Group allocation was randomized to minimize selection bias. However, blinding during and after surgery was not feasible. To ensure objective data interpretation, the analysis was conducted in a blinded manner. Sample size estimation was performed using G*Power (freely available software, Heinrich-Heine University Düsseldorf), applying a two-sided paired t-test with a significance level of 5% ($\alpha = 0.05$) and a statistical power of 80% ($1–\beta = 0.80$). The calculation was based on relevant data from the current literature. In accordance with the 3Rs principle (Replacement, Reduction, Refinement), the study was designed to begin with a minimal number of animals ($n = 3$ per group) for this feasibility phase, ensuring that no more animals than necessary were used. Behavioral tests were performed on days 1, 4, and 7 after surgery, not on the day of the procedure, to avoid confounding effects of anesthesia and minimize additional stress to the animals.

In vivo metabolism studies of (-)-[^{18}F]fluspidine

Blood and urine radio-metabolites were analyzed in 3 additional healthy female rats by analytical HPLC using the above-mentioned conditions, following an established protocol [*26*]. Each rat was injected via the lateral tail vein with 30 ± 5 MBq of (-)-[^{18}F]fluspidine in 1 mL of 0.9% NaCl solution under isoflurane anesthesia (1.5–2.5 vol% isoflurane with 2 L O_2/min). The animals remained anesthetized for 1 hour. Blood samples (400 µL) were collected at 5, 30, and 60 min postinjection via a freshly placed venous catheter. After the final blood collection, the rats were placed in metabolic cages and urine (500 µL) was collected within the first 5 minutes. On average, urine collection took 3.8 ± 0.7 minutes (mean ± SD, $n = 3$). This collection time was accounted for during subsequent calculations. Plasma was separated by centrifuging the blood samples at 10,000 rpm for 5 min. Plasma and urine samples were then prepared for reverse phase HPLC (RP-HPLC) analysis.

Protein precipitation of plasma and urine was carried out by adding ice-cold acetonitrile/water (ACN/H_2O, 9:1, v/v) in a 4:1 (v/v) ratio to the sample ($n = 3$). The samples were vortexed for 2 min, chilled on ice for 3 min, and centrifuged at 10,000 rpm at 4°C for 5 min. A second extraction was performed by washing the precipitates with 100 µL of the solvent mixture and repeating the procedure. The combined supernatants (total volume 100–200 µL) were collected and analyzed via analytical radio-HPLC under gradient conditions. Radioactivity levels in the supernatants and precipitates were measured using gamma counting to determine the percentage of total radioactivity recovered at each step. The animals were afterwards

internally transferred to a different study group in accordance with the 3Rs principles, following consultation with the animal facility and inspection by a veterinarian.

Surgical procedure and treatment

All surgeries were conducted under aseptic conditions following an established protocol [*27*]. General anesthesia was administered via isoflurane inhalation to all groups (induction: 5 vol% isoflurane with 5 L O_2/min; maintenance: 2 vol% isoflurane with 2 L O_2/min), accompanied by additional analgesia (carprofen, 4 mg/kg bw, s.c.; all groups) and antibiosis (cefuroxime,16 mg/kg bw, s.c.; only partial liver resection & skin incision groups). Blood samples were obtained during anesthesia from the vena sublingualis. Partial liver resection (50%) was conducted via laparotomy, involving a midline incision (~5 cm) from the xiphoid to the cranial pelvic brim after shaving and disinfection of the area. The respective lobes, including the left part of the medial liver lobe (LML), the left lateral lobe (LLL), and both caudate lobes (CL), were ligated with 4/0 silk (Resorba Medical GmbH, Nürnberg, Germany) at the hilus and excised with scissors [*27*] (Supplemental Fig. S1). To prevent further bleeding, the remaining stumps were cauterized with bipolar forceps (HF-Generator, MBC, Söring GmbH, Quickborn, Germany). Subsequently, the abdominal cavity was flushed with sterile saline (body temperature) to remove blood clots. After checking for any bleeding tissue, the muscle layer was closed with a two-layer continuous suture (5/0 Prolene, Johnson & Johnson Medical GmbH ETHICON, Norderstedt, Germany), while the skin was sutured with interrupted stitches (4/0 Vicryl, Johnson & Johnson Medical GmbH ETHICON). The skin incision group underwent a surgical cut at the same location without damaging the peritoneum, whereas the control group was only shaved in the respective area. After the intervention, the animals were placed in a pre-oxygenated, warmed intensive care unit (Vetario; Brinsea Products Ltd., North Somerset, UK) and received a single injection of sterile saline (10 mL/kg bw, s.c.). Postoperative analgesia (carprofen, 5 mg/kg bw, s.c.) and antibiotics (cefuroxime, 16 mg/kg bw, s.c.) were administered daily for 3 consecutive days, except for the control group, which received only analgesia.

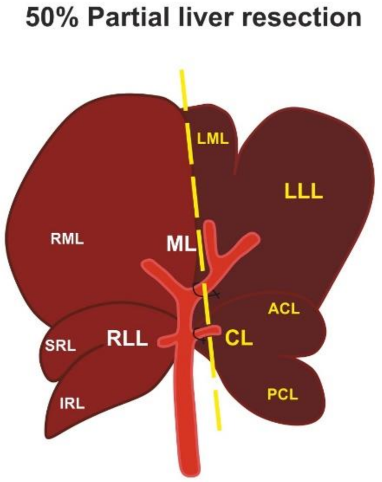

Supplemental Figure S1. Schematic representation of the liver lobes resected during partial (50%) liver resection. The respective lobes, including the left part of the medial liver lobe (LML), the left lateral lobe (LLL), and both caudate lobes (CL), were ligated with 4/0 silk at the hilus and excised with scissors. To prevent bleeding, the remaining stumps were cauterized with bipolar forceps. *LLL* left lateral lobe; *LML* left median lobe; *RML* right median lobe; *RLL* right liver lobes; *IRL* inferior right lobe; *SRL* superior right lobe; *CL* caudate lobes; *ACL* anterior caudal lobe; *PCL* posterior caudal lobe.

Open Field test

For measuring exploratory behavior and motor activity, rats were placed in a plastic box (L72 x W72 x H40 cm) with a dark underground. A 10 min video was recorded (Media Recorder 4, NOLDUS, Wageningen, The Netherlands; camera: Camera GigE monochrome, 1/1″; lens: Lens Std CS mount, 4.5–12.5 mm 1/2″, Basler AG, Ahrensburg, Germany) from above without additional adaption time [*27*]. Subsequently, the collected video footage was analyzed using NOLDUS EthoVision XT 14 software (Wageningen, The Netherlands) focusing on distance and velocity measurements.

Rat Grimace Scale

The Rat Grimace Scale (RGS) assesses pain by rating four categories (orbital tightening, nose/cheek flattening/tightening, ear position, whisker position) on a 3-point scale [*28*]. Rats were placed in polycarbonate observation boxes (L21 x W10.5 x H9 cm) and recorded for 10 min using a digital camera. Videos were semi-automatically analyzed according to the RGS [*29*]. Prior to RGS examination, observation boxes were placed in the rat's cage for a 5-min adaptation period.

Von Frey test

Hyperalgesia resulting from persistent pain was assessed using an Electronic Von Frey pressure algometer (BIO-EVF4, Bioseb, France) [*30*]. Rats were placed on a wire mesh floor, and their hind paw was stimulated with a 0.5 mm filament. The applied force was gradually increased until reaching the paw retraction threshold, recorded by the device (four repetitions). Potential hyperalgesia was determined by analyzing the withdrawal response to the mechanical stimuli.

Fecal corticosterone metabolite measurements

Fecal samples were collected during each intervention. Dried samples (50 mg) were dissolved in 80% methanol (Merck, Darmstadt, Germany), homogenized and centrifuged (10 min; 3000 g relative centrifugal acceleration, Fresco 21 & Pico 21 Heraeus, Hanau, Germany). Afterwards, fecal corticosterone metabolite concentrations were assessed by a 5α-pregnane 3β, 11β, 21-triol-20-one enzyme immunoassay [*31*].

PET/CT measurements

All animals were imaged using a small animal PET/SPECT/CT system (i.e., Triumph® II, Northridge Tri-Modality Imaging, Inc., Chatsworth, USA), however only the PET and CT modalities were used for this study. Each rat received an injection of 30 ± 5 MBq [^{18}F]fluspidine via lateral tail vein. After 1 hour of tracer incubation, a high-resolution CT scan (~ 15 min) was conducted, followed by a 20-minute PET scan. The following exposure settings were used: 130 uA, 75 kVp, 230 ms exposure time, and 360° rotation with 720 views with an average of two frames for each view. The CT had an axial field of view of 91.1 mm, and the PET had a field of view of 112 mm. During the scans, the isoflurane concentration was adapted to achieve a respiratory rate between 75–50 breaths per min.

Image processing and analysis

CT images were reconstructed using the Feldkamp filtered back projection method, resulting in a voxel size of 0.154 × 0.154 × 0.154 mm³ in a 592 × 592 × 560 matrix. CT values were converted to Hounsfield units (HU) using vendor-provided software and the following formula:

$$\mathrm{HU} = 1000 \text{ x } \frac{(\mu_t - \mu_w)}{\mu_w}$$

where μ_w is the linear attenuation coefficient of the water and μ_t is the linear attenuation coefficient of the tissue.

PET data were reconstructed using a 3D ordered-subset expectation maximization (OSEM-3D) with three iterations and eight subsets, combined with a maximum a posteriori probability

algorithm (30 iterations), producing a 240 × 240 × 192 image matrix (final voxel dimensions: 0.25 × 0.25 × 0.597 mm^3). PET normalization, CT attenuation correction, and CT scatter correction were applied to all reconstructions.

PET images were automatically aligned to the CT scans using a custom-made transformation based on a capillary phantom in the PMOD software package version 3.13 (PMOD Technologies LLC, Zürich, Switzerland). The co-registered PET/CT images were subsequently used for PET quantification.

The wound area in the abdominal wall was selected as the region of interest (ROI) for analysis. For user-independent segmentation, the cranial region of the T11 vertebral body was used as a reference, with a perpendicular line extending to the abdominal wall. From this slice, a cylindrical volume of interest (VOI) with a 2 mm radius and a 30 mm length was placed alongside the wound area as visualized on the CT. For control animals, this VOI was positioned along the *linea alba*. As a background reference, a 5 mm square was defined around the target ROI in the axial view and extended 30 mm to encompass the entire wound VOI. To exclude empty space outside the animal, both VOIs were initially reduced using CT-based automatic isocontouring with a minimal threshold of -600 HU. Due to the hepatobiliary clearance of (-)-[^{18}F]fluspidine, the VOIs were further reduced using PET data, applying the liver's mean activity serving as the maximum threshold. The mean uptake of the newly defined VOIs was recorded. All segmentation was carried out by a pseudo-blinded member of the team to minimize analysis bias.

To quantify the PET data and to correct for blood compartment contribution, the mean target-to-background ratio (TBRmean) was calculated using the following formula:

$$\mathrm{TBR_{mean}} = \frac{\mathrm{AverageUptake_{target}}}{\mathrm{AverageUptake_{background}}}$$

where $AverageUptake_{target}$ is the average in kBq/cc of the thresholded wound volume and $AverageUptake_{background}$ is the average in kBq/cc of the thresholded background volume surrounding the target.

Immunohistochemistry

Paraffin-embedded tissue slices (3−5 µm) were deparaffinized in xylene and permeabilized with PBS-T (0.1–0.2% Triton™ X-100 in PBS). σ1R expression was determined immunohistochemically using rabbit OPRS1 Polyclonal Antibody (1 µg/µL; Bioss antibodies), followed by goat-anti-rabbit IgG (H&L)-HRP (0.5 mg/mL; Abcam) and DAPI (Merck). Signal amplification was performed using TSA™ Plus Cyanine 5 (Perkin Elmer/Akoya Biosciences®) detection kit. Apoptotic cells were stained using the *in situ* cell death detection kit (TUNEL,

Roche, Basel, Switzerland). During fluorescence microscopy, five randomly selected visual fields were analyzed per slice. Signal-positive cells were quantified using Fiji (ImageJ2) to calculate the percentage of $σ1R^+$ and apoptotic cells relative to $DAPI^+$ cells.

Blood parameter analysis

Blood samples were obtained from the sublingual vein during surgery and from the caval vein of anesthetized animals on the last day. EDTA blood samples were analyzed using the Celltac α MEK-6450 K analyzer (Nihon Kohden Europe GmbH, Rosbach vor der Höhe, Germany).

Statistical analysis

Data were tested for normality and analyzed with one-way or two-way ANOVA, followed by a Tukey or Dunnett post hoc test on a 95% confidence interval (SPSS, IBM Corp, v25, Sanborn, NY, academic license; GraphPad Prism5, v5.01, San Diego, CA, academic license). Statistical significance was determined by a probability (p) value of <0.05. Means ± standard deviations are presented.

Supplemental Tables

Supplemental Table S1. [^{18}F]Fluspidine autoradiography blocking study. Detailed results on tracer uptake [prop.to.cnts/sec] in healthy rat brains. All results are reported as means ± standard deviations.

Parameter	[^{18}F]Fluspidine	Fluspidine Block	Haloperidol Block
Tracer uptake [prop.to.cnts/sec]	1733.1 ± 153.6	1436.3 ± 107.0	1395.9 ± 123.7
Tissue slices *n* = 9/ substance			

Supplemental Table S2. [^{18}F]Fluspidine *in vivo* metabolism study. Detailed results on tracer concentrations in blood and urine at 5-, 30-, and 60-min post-injection.

	Rat 1				Rat 2				Rat 3			
	Blood samples			Urine sample	Blood samples			Urine sample	Blood samples			Urine sample
**Time point (min)**	**5**	**30**	**60**	**>60**	**5**	**30**	**60**	**>60**	**5**	**30**	**60**	**>60**
Recovery [%]	84.0	52.0	41.0	Not needed	79.0	55.0	50	Not needed	65	59	61	Not needed
Radiotracer [%]	*7.2**	7.5	<8	2.3	46.5	11.2	<8	1.1	30.6	12.9	<8	1.0

Recovery [%] based on gamma counting; radiotracer [%] based on RP-HPLC after recovery correction a
*Measurement error

Supplemental Table S3. PET/CT quantification and immunohistochemical (IHC) validation of [^{18}F]fluspidine uptake at incision sites on postoperative day 1-7. Detailed information on radiotracer uptake and IHC staining for σ1 receptor positive (σ1R^{+}) and apoptotic cells at the incision site. All results are reported as means ± standard deviations.

Parameter	Postoperative Day 1			Postoperative Day 4			Postoperative Day 7		
	**Control**	**Skin incision**	**Liver resection**	**Control**	**Skin incision**	**Liver resection**	**Control**	**Skin incision**	**Liver resection**
TBRmean [Target/ Background]	1.1 ± 0.1	1.0 ± 0.1	1.8 ± 0.2	0.9 ± 0.1	1.0 ± 0.1	1.0 ± 0.1	1.0 ± 0.1	1.0 ± 0.1	1.0 ± 0.1
σ1R^{+} cells [%]	0.2 ± 0.1	1.2 ± 0.6	9.0 ± 0.6	0.2 ± 0.1	0.4 ± 0.2	1.2 ± 0.2	0.2 ± 0.1	0.2 ± 0.1	0.3 ± 0.1
TUNEL^{+} cells [%]	0.2 ± 0.1	0.8 ± 0.2	1.8 ± 0.4	0.4 ± 0.1	0.5 ± 0.1	0.9 ± 0.2	0.4 ± 0.1	0.5 ± 0.1	0.6 ± 0.1
Data collected: *n* = 3/ group/ postoperative day 1,4 or 7 (euthanisation)									

Supplemental Table S4. Daily postoperative clinical score sheet monitoring in rats. Alterations in bodyweight, general state, spontaneous behavior, and surgery specific parameters were documented and allocated to a point grading system. No alterations in physiological state were graded as 0 points, whereas ≥ 20 points marked the highest severity and humane endpoint.

**Parameters for postoperative monitoring in rats**	
**Observation**	**Score**
**I Bodyweight**<br>• No changes or increase in weight<br>• Weight reduction 1 – 5 %<br>• Weight reduction 5 – 10 %<br>• Weight reduction 11 – 19 %<br>• Weight reduction ≥ 20 %	 0<br>1<br>5<br>10<br>20
**II General condition**<br>• Shiny fur, clean body openings, clear and shiny eyes<br>• Defects of the fur, (increased or decreased body care)<br>• Dull fur, untidy, unclean body openings, cloudy eyes, increased muscle tonus<br>• Dirty fur, clotted and soggy body openings, unusual posture, dull eyes, high muscle tonus, diarrhea (<48 h)<br>• Cramps, paralysis (trunk muscles, extremities), conspicuous breathing problems, cold body	 0<br>1<br>5<br>10<br><br>20
**III Spontaneous behavior**<br>• Normal behavior (sleep, reaction to blowing and touching, curiosity, social contacts)<br>• Slight changes to normal behavior, 'pain' relieving posture<br>• Unusual behavior, impaired/reduced motor function<br>• Self-isolation, lethargy, reduced pronounced behavioral stereotypes, coordination disorders<br>• Pain sounds when seizing, self-amputation (auto aggression, autotomy), apathy, noticeable defensive reactions	 0<br>1<br>5<br>10<br><br>20
**IV Surgery specific parameters**<br>• Unreactive surgery wound, no swelling<br>• Surgery wound is slightly red, gnawing/manipulation of suture ends (wound closed)<br>• Reddening of the surgery wound, slight swelling of the wound, wound manipulation<br>• Surgery wound with wound secretion, pronounced gnawing/manipulation of the wound, slight wound infection, suture dehiscence, light bleeding, swelling of the tongue<br>• Repetitive suture dehiscence, severe wound infection, hemoperitoneum, severe swelling of the tongue with open mouth, impaired breathing,	 <br>0<br>1<br>5<br>10<br><br>20
**Assessment**	**Total score**
• **Degree of burden 0**: no burden<br>• **Degree of burden 1:** mild burden; continue to observe carefully<br>• **Degree of burden 2:** moderate burden; if necessary, initiate veterinary care (analgesia; in case of suture dehiscence, renewed wound care under anesthesia)<br>• **Degree of burden 3:** severe burden; **humane endpoint:** immediate termination of the experiment and euthanasia	0<br>1 – 9<br>10 – 19<br><br>>20

Supplemental Table S5. Postoperative pain monitoring by physiological parameters. Detailed results of animal welfare assessment parameters (clinical score sheet, bodyweight, Open Field test: distance and velocity, Von Frey test: Paw withdrawl threshold, Rat Grimace Scale, Fecal corticosterone metabolite levels) of rats on postoperative day 1-7. FCMs, Fecal corticosterone metabolites; RGS, Rat Grimace Scale. All results are reported as means ± standard deviations.

	**Postoperative Day 1**			**Postoperative Day 4**			**Postoperative Day 7**		
**Parameter**	**Control**	**Skin incision**	**Liver resection**	**Control**	**Skin incision**	**Liver resection**	**Control**	**Skin incision**	**Liver resection**
Score points	0.2 ± 0.4	1.7 ± 1.8	0.6 ± 0.7	0 ± 0	0.3 ± 0.5	2.2 ± 2.0	0 ± 0	0.7 ± 0.5	1.0 ± 0
Bodyweight [g]	211.4 ± 12.5	210.9 ± 6.1	208.7 ± 11.4	215.0 ± 15.6	213.0 ± 8.6	207.5 ± 11.6	213.7 ± 9.5	217.3 ± 3.4	214.3 ± 10.3
Open Field distance [cm]	4732.3± 1119.4	4301.3± 1465.8	4889.0± 686.0	4547.8± 877.4	5510.3± 175.9	5009.0± 805.2	4996.4± 680.9	5843.0± 282.2	6597.9± 1038.2
Open Field velocity [cm/min]	498.8 ± 117.5	532.2 ± 100.9	517.5 ± 97.0	472.2 ± 94.6	631.1 ± 12.5	562.8 ± 108.5	519.8 ± 79.7	611.2 ± 25.8	518.3 ± 73.6
VFT Withdrawl threshold [g]	59.1 ± 8.4	58.2 ± 4.1	58.0 ± 9.1	48.0 ± 8.3	44.5 ± 5.5	31.7 ± 5.8	56.6 ± 7.1	48.3 ± 7.8	54.9 ± 5.0
RGS score points	1.5 ± 0.1	1.4 ± 0.2	1.4 ± 0.3	1.2 ± 0.1	1.4 ± 0.3	2.3 ± 0.5	1.9 ± 0.4	1.5 ± 0.2	2.0 ± 0.4
FCMs [µg/g feces]	3.5 ± 0.8	5.5 ± 2.6	6.7 ± 2.5	5.8 ± 2.0	5.8 ± 1.1	4.2 ± 2.0	6.7 ± 1.8	6.0 ± 3.8	3.6 ± 1.1

Data collected: *n* = 3/ group/ postoperative day 1,4 or 7 (euthanisation)

Supplemental Table S6. Hemogram results on surgery day and euthanisation day. Detailed results from hemogram analysis (leukocyte-, erythrocyte-, thrombocyte counts, hemoglobin, hematocrit) of all groups. All results are reported as means ± standard deviations.

	Surgery			Postoperative Day 1			Postoperative Day 4			Postoperative Day 7		
**Parameter**	**Control**	**Skin incision**	**Liver resection**	**Control**	**Skin incision**	**Liver resection**	**Control**	**Skin incision**	**Liver resection**	**Control**	**Skin incision**	**Liver resection**
Leukocytes [10^3/µL]	9.9 ± 1.6	9.3 ± 2.0	9.1 ± 1.7	5.7 ± 1.7	4.2 ± 1.3	4.1 ± 0.6	3.8 ± 0.7	4.7 ± 0.7	4.8 ± 0.5	3.8 ± 0.8	3.5 ± 0.2	4.5 ± 0.3
Erythrocytes [10^6/µL]	7.9 ± 0.5	8.0 ± 0.4	7.8 ± 0.4	7.1 ± 0.5	7.2 ± 0.5	6.3 ± 0.8	7.5 ± 0.5	6.9 ± 0.4	7.1 ± 0.3	7.3 ± 0.6	7.3 ± 0.7	6.9 ± 0.3
Thrombocytes [10^3/µL]	860.8 ± 115.9	972.3 ± 122.3	832.8 ± 240.3	914.3 ± 188.1	1064.3± 162.0	804.7 ± 116.4	910.3 ± 152.4	1088.3± 95.3	919.7 ± 39.1	767.0 ± 140.9	935.7 ± 108.8	582.7 ± 349.9
Hemoglobin [g/dL]	15.6 ± 1.1	15.7 ± 1.1	15.1 ± 0.9	14.1 ± 1.1	14.4 ± 0.9	12.4 ± 1.2	14.5 ± 1.2	13.1 ± 0.8	14.0 ± 1.1	14.2 ± 0.8	14.0 ± 0.4	13.5 ± 0.4
Hematocrit [%]	43.5 ± 1.9	43.6 ± 1.8	42.6 ± 2.2	41.2 ± 3.4	41.1 ± 2.4	35.4 ± 3.5	42.3 ± 3.7	39.2 ± 1.9	39.2 ± 2.5	40.4 ± 1.9	38.9 ± 1.1	32.2 ± 9.9

Data collected: day 0 (surgery) *n* = 27 and *n* = 3/ group/ postoperative day 1,4 or 7 (euthanisation)

Supplemental Figures:

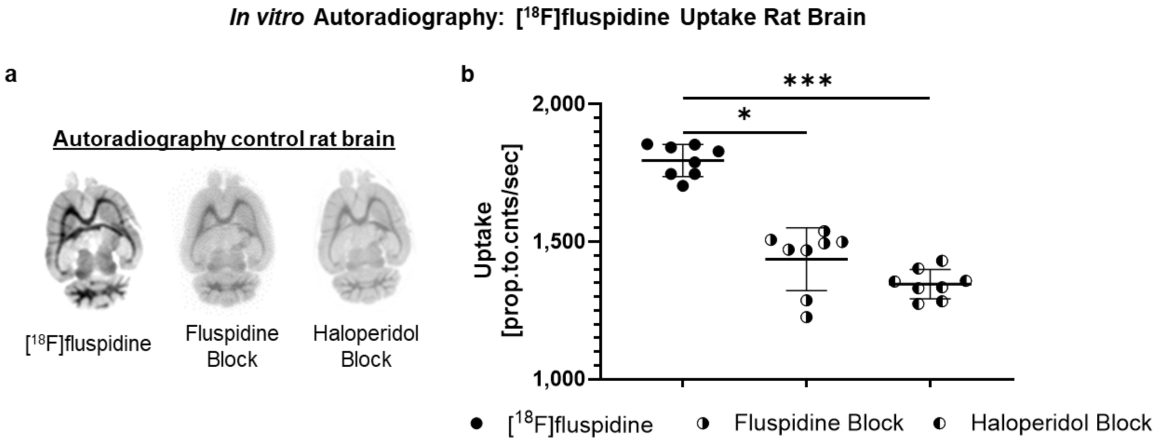

Supplemental Figure S2. [18F]Fluspidine autoradiography blocking study. (**a-b**) The radiotracer uptake in pre-blocked (haloperidol 10 µM or fluspidine 10 µM) rat brain slices is significantly lower than in non-blocked slices. n = 9; **p<0.01, ***p<0.0005.

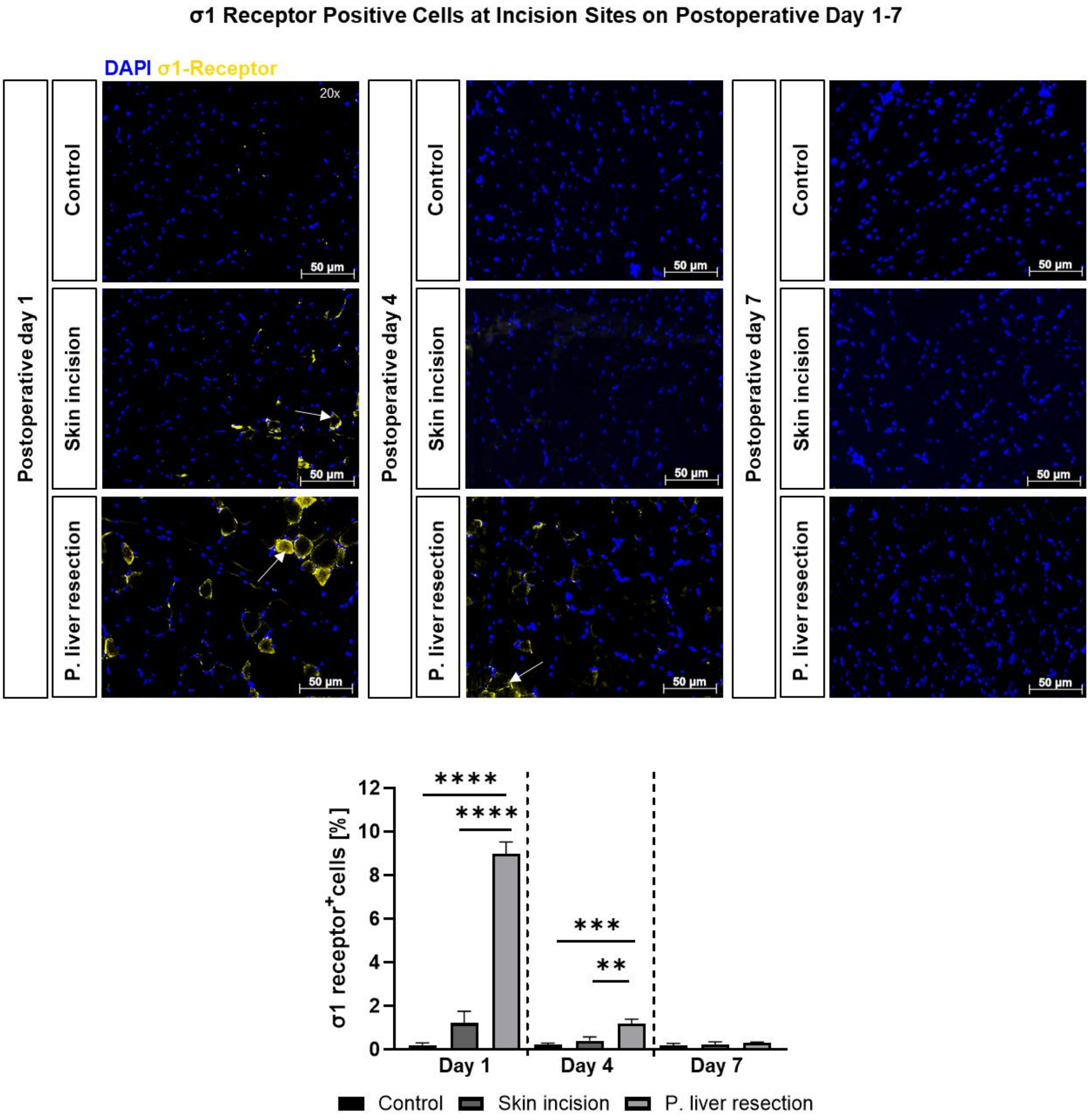

Supplemental Figure S3. Immunohistochemical (IHC) staining of the σ1 receptor at incision sites (skin and muscle) on postoperative day 1-7. Significantly more σ1 receptor positive cells (white arrow) are detected by IHC staining at the incision sites after liver resection compared to the control and skin incision group on postoperative day 1. On postoperative day 4, only a few residual σ1 receptor positive cells can be observed in liver resected rats. Comparable numbers of σ1 receptor positive cells can be detected on postoperative day 7 in all groups. Results are presented as means ± standard deviations. Scale bar: 50 µm (images taken at 20x magnification). *P*. partial. **$p < 0.01$, ***$p < 0.0005$, ****$p < 0.0001$.

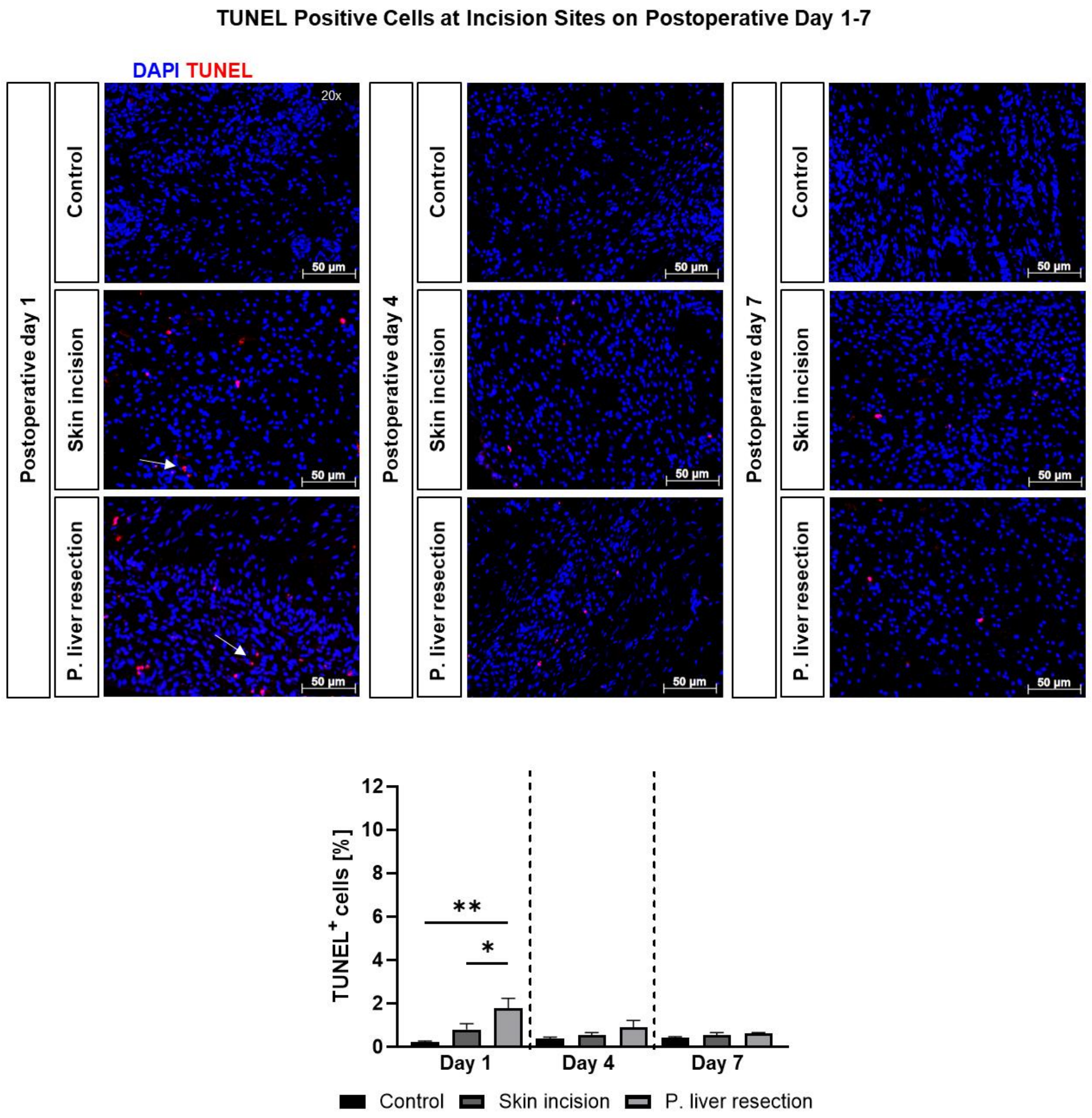

Supplemental Figure S4. Immunohistochemical (IHC) staining of apoptotic cells at incision sites (skin and muscle) on postoperative day 1-7. Significantly more TUNEL positive cells (white arrow) are detected by IHC staining at the incision sites after liver resection compared to the control and skin incision group on postoperative day 1. Comparable numbers of apoptotic cells can be detected on postoperative day 4 and 7 in all groups. Results are presented as means ± standard deviations. Scale bar: 50 µm (images taken at 20x magnification). *P.* partial. *$p < 0.05$, **$p < 0.01$.

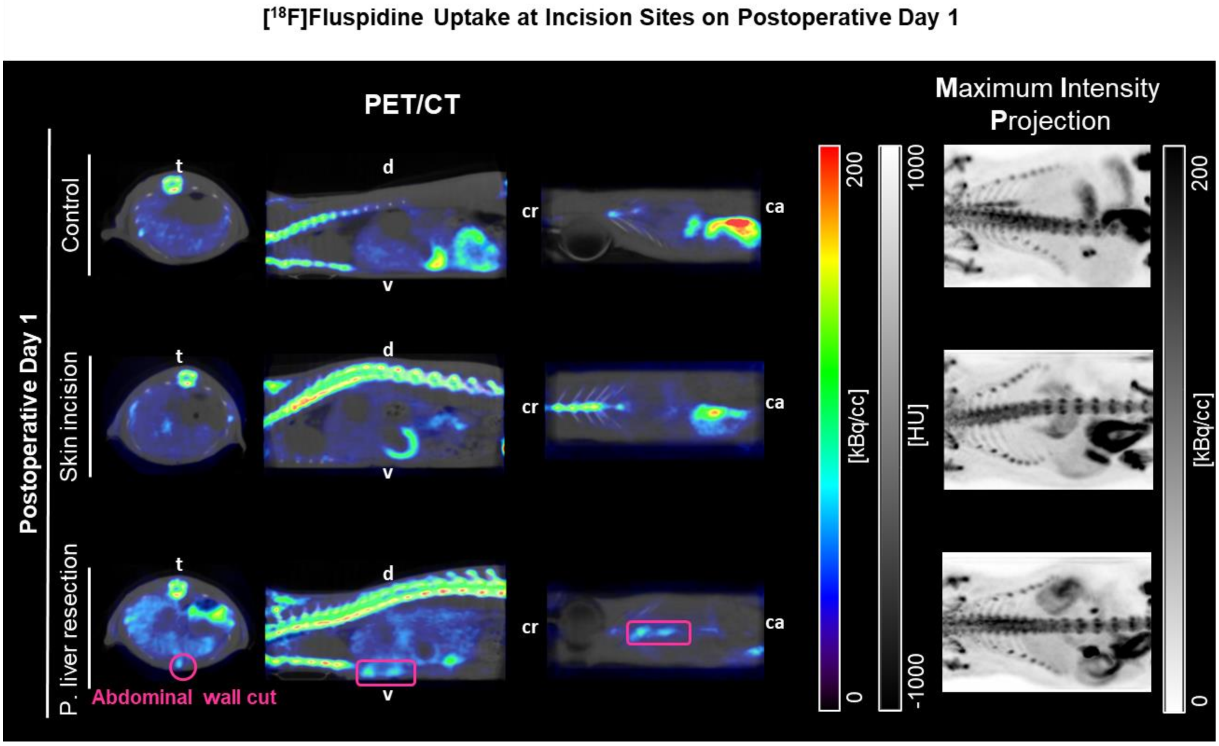

Supplemental Figure S5. PET/CT images of [^{18}F]fluspidine uptake at incision sites on postoperative day 1. PET/CT images show a significantly higher radiotracer uptake at the incision sites after partial liver resection compared to the other groups. Free fluorine is observed in the spine and ribs. PET/CT imaging was performed 60 min post-injection. *ca* caudal; *cr* cranial; *d* dorsal; *P*. partial; *t* transversal; *v* ventral.

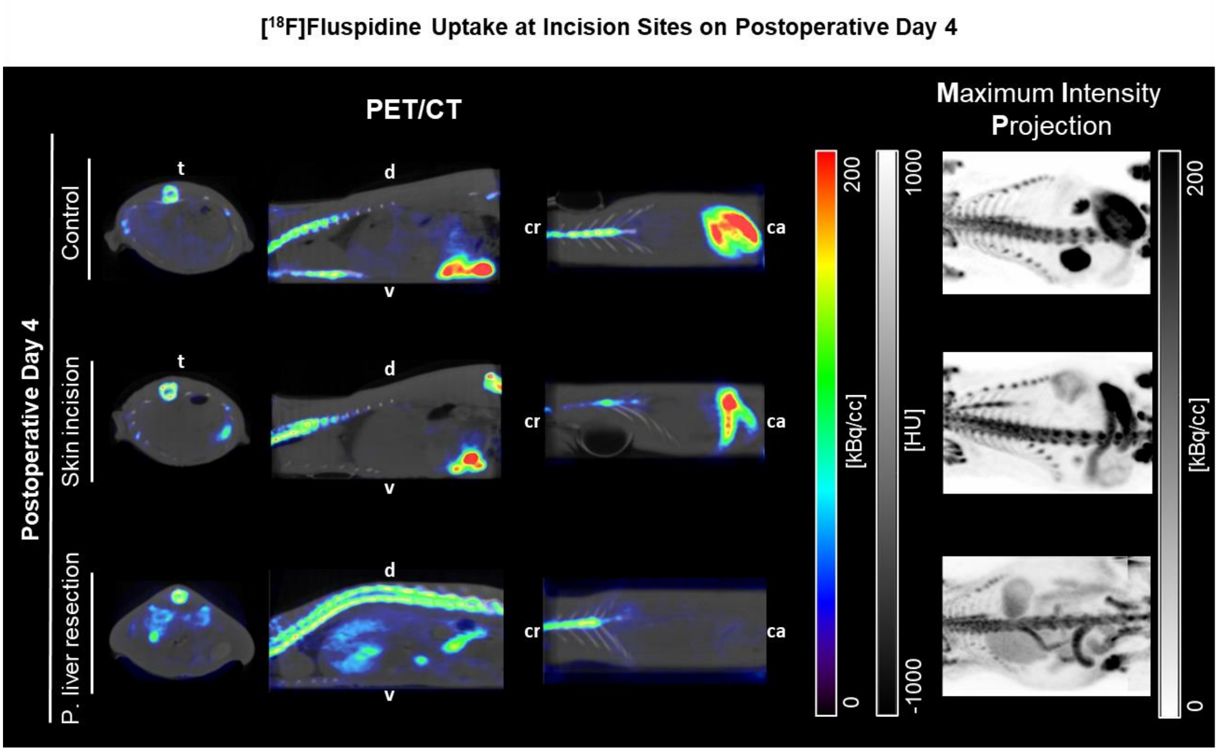

Supplemental Figure S6. PET/CT images of [^{18}F]fluspidine uptake at incision sites on postoperative day 4. PET/CT images show no significant radiotracer uptake at the incision sites after surgery on postoperative day 4. Free fluorine is observed in the spine and ribs. PET/CT imaging was performed 60 min post-injection. *ca* caudal; *cr* cranial; *d* dorsal; *P*. partial; *t* transversal; *v* ventral.

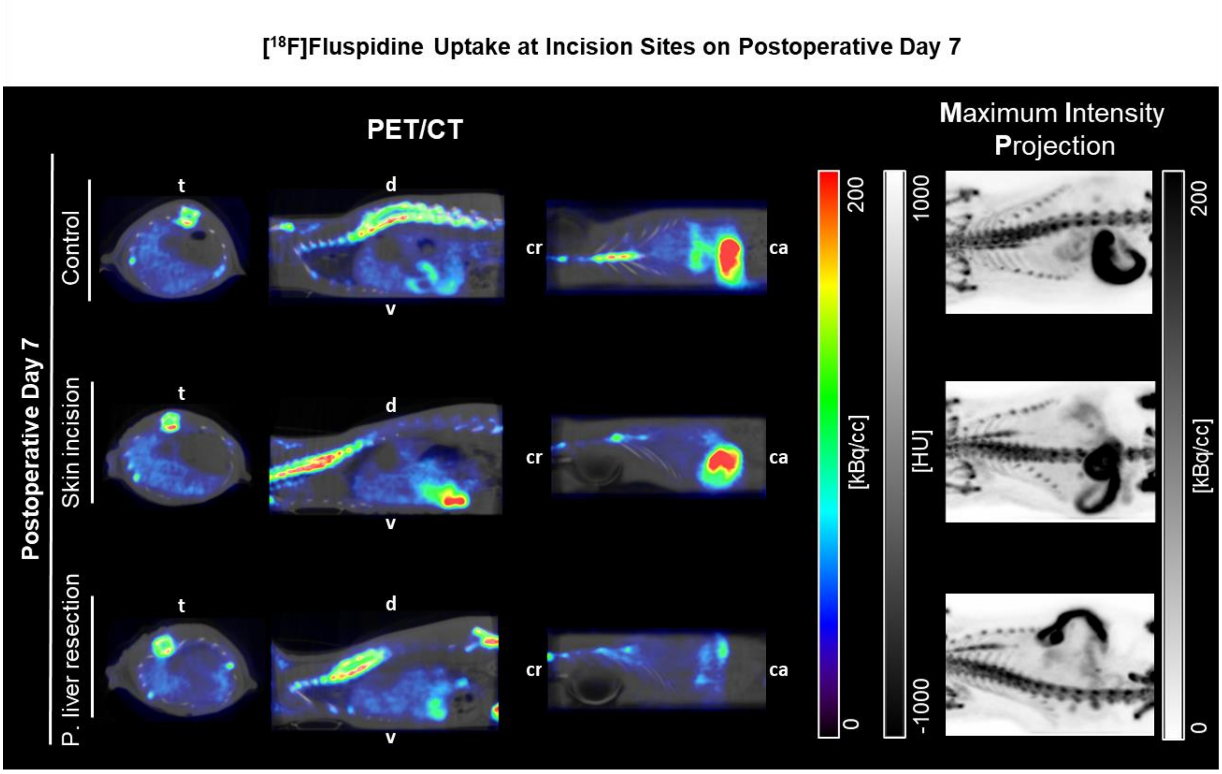

Supplemental Figure S7. PET/CT images of [18F]fluspidine uptake at incision sites on postoperative day 7. On postoperative day 7 no significant radiotracer uptake can be detected at the incision sites by PET/CT imaging. Free fluorine is observed in the spine and ribs. PET/CT imaging was performed 60 min post-injection. *ca* caudal; *cr* cranial; *d* dorsal; *P*. partial; *t* transversal; *v* ventral.

Leukocyte Counts before Surgery and on Postoperative Day 1-7

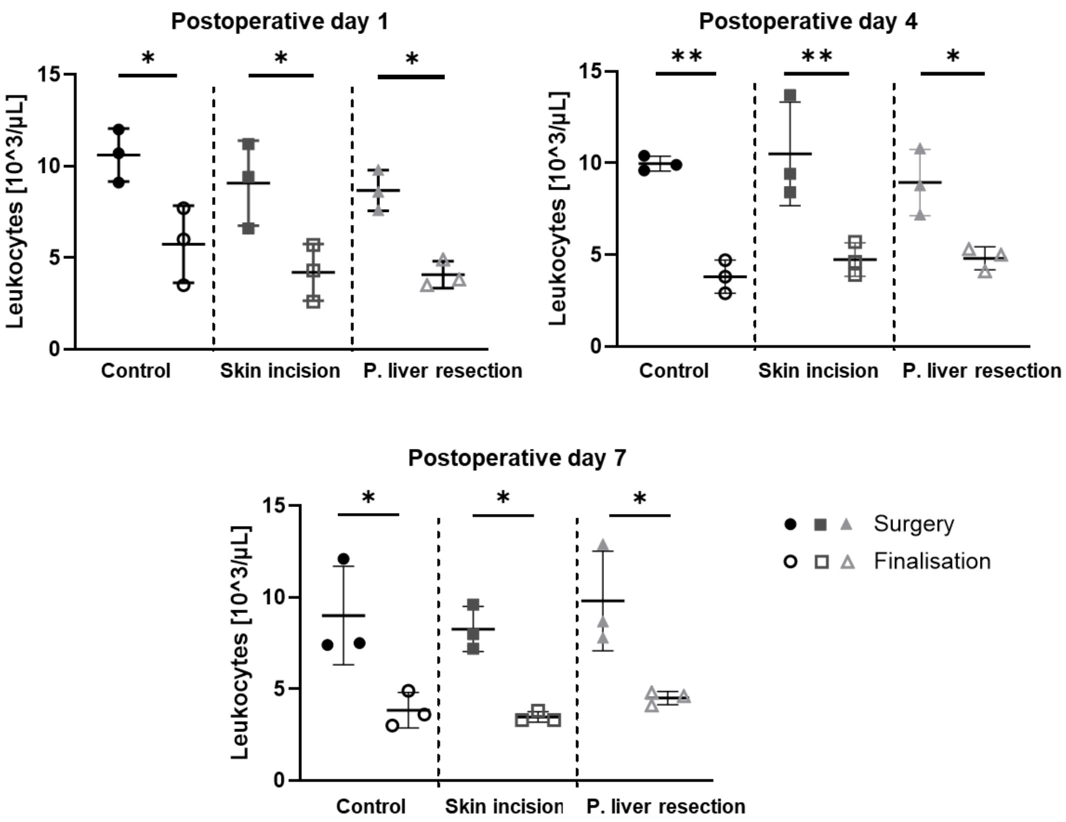

Supplemental Figure S8. Leukocyte counts before surgery and on postoperative day 1-7. A significant decrease in leukocyte counts can be observed across all groups on the final day of the experiment (2 hours after [^{18}F]fluspidine tracer injection), independent of the postoperative day or surgical intervention. *P* partial. **p* < 0.05, ***p* < 0.01.
